# Maternal topoisomerase II alpha, not topoisomerase II beta, enables embryonic development of zebrafish *top2a^-/- ^*mutants

**DOI:** 10.1186/1471-213X-11-71

**Published:** 2011-11-23

**Authors:** Beata Sapetto-Rebow, Sarah C McLoughlin, Lynne C O'Shea, Olivia O'Leary, Jason R Willer, Yolanda Alvarez, Ross Collery, Jacintha O'Sullivan, Freek Van Eeden, Carmel Hensey, Breandán N Kennedy

**Affiliations:** 1 UCD School of Biomolecular and Biomedical Science, UCD Conway Institute, University College Dublin, Dublin, Ireland; 2 Department of Biochemistry and Molecular Biology, University of Louisville, Louisville, KY, USA; 3 St. James's Hospital, Trinity College Dublin, Dublin, Ireland; 4 Department of Biomedical Science, University of Sheffield, Sheffield, UK; 5 Institute for Translational Medicine and Therapeutics, School of Medicine, University of Pennsylvania, Philadelphia, PA, USA; 6 Department of Cell Biology, Neurobiology & Anatomy, Medical College of Wisconsin, Milwaukee, WI, USA

## Abstract

**Background:**

Genetic alterations in human topoisomerase II alpha (*TOP2A*) are linked to cancer susceptibility. TOP2A decatenates chromosomes and thus is necessary for multiple aspects of cell division including DNA replication, chromosome condensation and segregation. Topoisomerase II alpha is also required for embryonic development in mammals, as mouse *Top2a *knockouts result in embryonic lethality as early as the 4-8 cell stage. The purpose of this study was to determine whether the extended developmental capability of zebrafish *top2a *mutants arises from maternal expression of *top2a *or compensation from its *top2b *paralogue.

**Results:**

Here, we describe *bloody minded (blm)*, a novel mutant of zebrafish *top2a*. In contrast to mouse *Top2a *nulls, zebrafish *top2a *mutants survive to larval stages (4-5 day post fertilization). Developmental analyses demonstrate abundant expression of maternal *top2a *but not *top2b*. Inhibition or poisoning of maternal topoisomerase II delays embryonic development by extending the cell cycle M-phase. Zygotic *top2a *and *top2b *are co-expressed in the zebrafish CNS, but endogenous or ectopic *top2b *RNA appear unable to prevent the *blm *phenotype.

**Conclusions:**

We conclude that maternal *top2a *enables zebrafish development before the mid-zygotic transition (MZT) and that zebrafish *top2a *and *top2b *are not functionally redundant during development after activation of the zygotic genome.

## Background

Topoisomerase (DNA) II alpha (TOP2A) is a nuclear protein which regulates DNA architecture during the mitotic phase of the cell cycle [1]. Studies in *Xenopus *have shown that its function in chromatin condensation is tightly coupled to prior DNA replication [2-4]. The expression of *TOP2A *is cell cycle regulated, reaching a peak in the G2/M phase [5]. Thus, up-regulated levels of TOP2A protein are found in proliferating cancer cells, and TOP2A is essential for the viability of these dividing cells. Consistent with its role in cell proliferation, genetic aberrations in *TOP2A *are linked to numerous human cancers [6]. To facilitate proper separation of chromatids and DNA replication, TOP2A generates transient double-stranded breaks in DNA [7-10]. Pharmacological targeting of TOP2A, which is extensively applied in cancer treatment, exploits this mechanism [11].

Vertebrate genomes contain two topoisomerases II paralogues. Human TOP2A and TOP2B have similar molecular masses (180 and 170 kDa, respectively) and share ~70% amino acid similarity, with the greatest divergence occurring in the C-terminal domain [12]. Despite structural similarities, these topoisomerases II isoforms have different expression patterns and functions [13]. In contrast to the expression of mammalian Top2a which peaks at G2/M, mammalian Top2b is expressed in differentiated tissues and its expression is not cell cycle regulated [14-17]. Several *in vitro *models have been utilised to study the *loss-of-function *effects associated with topoisomerase II genes. However, the critical role of *Top2a *genes for cell proliferation and survival necessitates conditional knockout models [18,19]. In vivo, genetic elimination of *Top2a *is dramatic, with mouse knockouts not developing beyond the 4-8 cell stage [20]. Underlying a distinct role, *Top2b *knockouts are not embryonic lethal. Instead *Top2b *is required during neuronal differentiation, for survival of some neural cells and neurite outgrowth [9,21,22]. *Top2b *null mice have defects in cerebral stratification and motor axons fail to contact skeletal muscles resulting in death soon after birth due to breathing impairment [23,24]. The topoisomerases II paralogues are also important as targets of anti-cancer drugs. ICRF-193, a catalytic inhibitor, blocks topoisomerase II-mediated ATP hydrolysis which is required to regenerate its active enzymatic form [25]. Etoposide, a topoisomerase II poison, inhibits the ability of topoisomerase II to re-ligate DNA molecules and therefore stabilises cleavable complexes of the enzyme leading to extensive fragmentation of DNA and cell death [26]. Topoisomerase II poisons are reported to mediate their cytotoxic effects through TOP2A, while double strand breaks and DNA rearrangements associated with secondary malignancies are due to TOP2B [27].

Two *top2a *mutant alleles have previously been documented in zebrafish: *hi3635 *generated by viral insertion [28] and *can4 *by ENU mutagenesis [29]. These mutants present with similar phenotypes including brain necrosis, abnormal tail curvature and death at 4-5 dpf. The *can4 *mutants have been studied more thoroughly and display reduced cell proliferation, mitotic spindle defects and increased DNA content. Recently, a zebrafish *top2b *mutant was reported with a phenotype distinct to *top2a *mutants, including defects in neurite targeting within the retinal inner plexiform layer and tectal neuropil [30]. These distinct phenotypes indicate separate functions of the zebrafish Top2a and Top2b isoforms.

Previous studies, however, do not explain the viability of zebrafish *top2a *mutants up to larval stages, in contrast to the early embryonic lethality of mouse *Top2a *knockouts. One explanation could relate to differential requirements of teleost and mammalian embryos for the timing of initiation of zygotic transcription, *i.e*. transcription from the embryo's own genome. The mid-blastula transition (MBT) refers to the moment during the blastula stage of embryonic development when expression of the zygotic genome starts, cell cycles lengthen, and cells acquire the ability to migrate [31]. The original term MBT (still used in relation to *Xenopus*) was expanded to include a phase of elimination of maternal transcripts and proteins starting before the activation of zygotic transcription [32]. This developmental event is referred as maternal-to-zygotic-transition (MZT) in recent studies [33]. In zebrafish, zygotic transcription increases gradually from the 10^th ^(~2.75 hpf) to 13^th ^cycle (~4.75 hpf) [34,35].

Here, we describe a novel zebrafish *top2a *mutant named *"bloody minded" *(*blm*) which was identified in an ENU mutagenesis screen. Mutant larvae can survive to ~5 dpf, although abnormal embryo morphology is discernable at ~27 hpf. We tested the hypotheses that the advanced development of zebrafish *top2a *mutants is due to either the presence of maternal Top2a or redundancy with the Top2b isoform. Our results are consistent with early embryonic development depending on maternal transcripts of zebrafish *top2a*. The absence of zygotic *top2a *is not fully compensated for by maternal, zygotic or ectopic *top2b *mRNA suggesting distinct functions of Top2a and Top2b in embryonic development.

## Results

### *blm *is a lethal recessive zebrafish mutant

**Figure 1:**
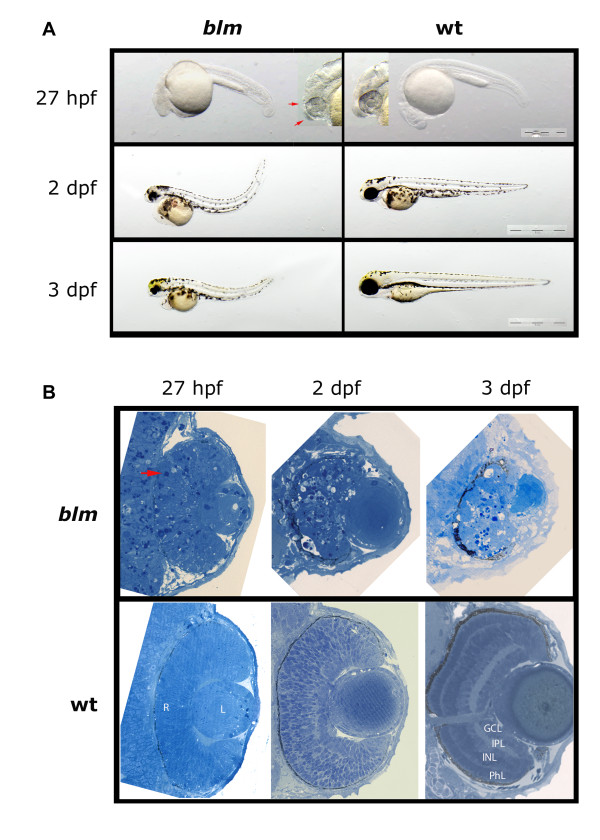
**Phenotype of the zebrafish *bloody minded *(*blm*) mutant**. **A) **Mutants can be first recognized at ~27 hpf when signs of malformation in the head are visible (red arrows). At 2 and 3 dpf, eyes are much smaller and the tail is bent dorsally. **B) **Plastic sections through the eye reveal apoptotic cells in the retina (red arrow) and in the brain at 27 hpf. There is no proper lamination of the retina and necrosis proceeds. Abbreviations: hpf - hours post fertilisation, dpf - days post fertilisation, R - retina, L - lens, PhL - photoreceptor layer, INL - inner nuclear layer, IPL - inner plexiform layer, GCL - ganglion cells layer.

During F3 mutagenesis screens we recovered a recessive-lethal mutant named *bloody-minded *(*blm*). The *blm *phenotype is first visible at ~27 hpf when forebrain and midbrain atrophy is apparent and occasionally accompanied with CNS haemorrhage (Figure 1A). Increased cell death is prominent in the *blm *head, retina and lens at ~27 hpf (Figure 1B). The eyes fail to develop further so that at 2-3 dpf, *blm *eyes are significantly smaller than wildtype siblings and *blm *lenses protrude from the eye. At this stage, *blm *retinae fail to properly laminate and dying cells are visible. The body axes of *blm *mutants curves dorsally, cardiac oedema develops and *blm *mutants die at 4-5 dpf.

### *blm *arises from a nonsense mutation in the *top2a *gene

**Figure 2:**
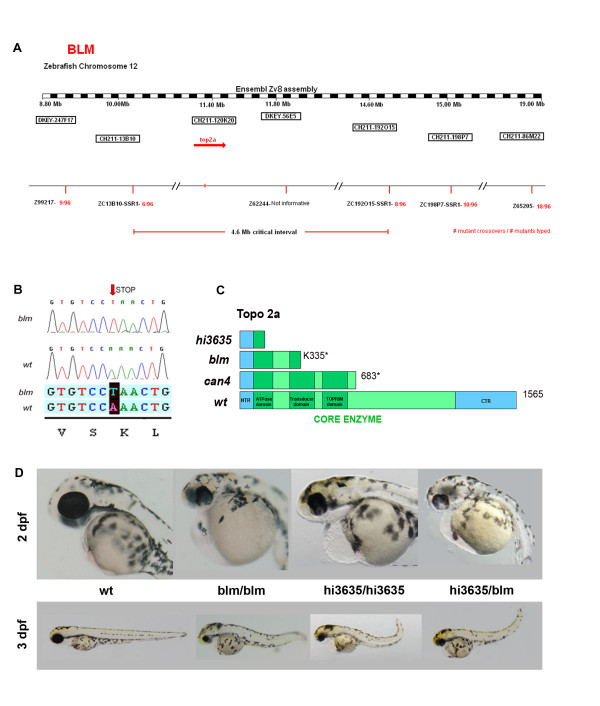
***blm *arises from a mutation in the *top2a *gene**. **A) **Mapping of *blm *reveals that the gene is located on chromosome 12. **B) ***top2a *was selected as a candidate gene and sequencing of mutant cDNA shows a point mutation (A→T) in Lys residue 335 resulting in a premature stop codon. **C) **A schematic of wildtype Top2a protein and the truncated proteins in *hi3635*, *blm *and *can4 *mutants **D) **Complementation assay performed to confirm that *blm *is *top2a *mutation. *blm *heterozygotes were crossed to *hi3635 *carriers and the resulting hybrid offspring present with *blm *phenotype.

To determine the genetic locus of the *blm *mutation we performed bulk segregant analysis. Linkage to the *blm *phenotype was found on chromosome 12 with flanking Z-markers Z99217 and Z10806 (Figure 2A). Several new simple sequence repeat (SSR) markers were designed to narrow the critical interval of which zC13B10-SSR1 was most closely linked (6/96 recombinants). Analyses of the genetic maps in this interval revealed that a viral insertion mutant *hi3635*, with gross morphology similar to *blm*, co-segregated with the *blm *interval [28]. As *hi3635 *arises from an insertion in exon 1 of the gene encoding topoisomerase IIa (*top2a*), we chose *top2a *as a candidate gene for *blm*. Sequencing of whole larval cDNA from *blm *mutants reveals a point mutation (A→T) which introduces a premature stop codon at Lys residue 335 (K335X) (Figure 2B). The resulting truncated protein lacks the enzymatic domain (Figure 2C).

To confirm that the *blm *phenotype arises from a mutation in the *top2a *gene we performed complementation assays (Figure 2D). Matings of *blm *carriers and *hi3635 *carriers fail to complement. Matings of *blm*^+/- ^with *hi3635*^+/- ^generated ~30% embryos with the *blm *phenotype, whereas outcrosses to wildtype fish did not. Thus, genomic and genetic data confirm that the *blm *mutant results from a nonsense mutation in the zebrafish *top2a *gene.

### *top2a *mutants exhibit defects in cell cycle progression

**Figure 3:**
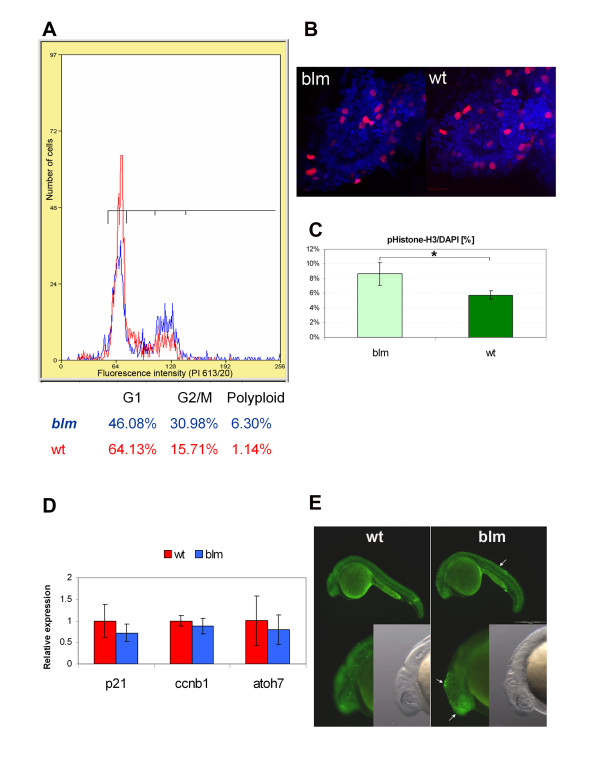
***top2a *mutants exhibit defects in cell cycle progression**. **A) **Flow cytometry analysis of larvae at 27 hpf (red plot - wt, blue - *blm*) reveals an increased fraction of cells in G2/M phase in *blm *mutants. **B) **Representative Z-series projections showing that the total number of mitotic cells (stained with PH3 antibody - red) is lower in the eye of 27 hpf *blm *mutants (n = 58) than in wild type larvae (n = 70). **C) **The percentage of mitotic cells (PH3 positive) relative to the total number of DAPI stained cell nuclei is significantly (p = 0.036) higher in *blm *mutants compared to wild type as assessed from n ≥ 23 sections through the head and spinal cord of the N = 5 mutant and wild type larvae. **D) **Real time PCR of cell cycle markers *p21-like *(G1 phase) and *ccnb1 *(G2/M phase) in wildtype and *blm *larvae at 27 hpf shows no significant difference in expression levels (p = 0.21 and 0.38, respectively). Expression of *atoh7*, a marker of retinogenesis, is also equivalent in eyes of *blm *and wildtype siblings. Presented data are average of 3 replicate experiments, each comprising pools of 16-35 larvae. Error bars represent the standard error of the mean (C-D). **E) **Acridine orange staining of apoptotic cells in wildtype and *blm *larvae at 24 hpf. Arrows point to regions of increased apoptosis in *blm *mutants.

Topoisomerase II alpha genes are known to be critical for cell cycle progression. Therefore, we quantified, by flow cytometry, the distribution of *blm *cells within the major phases of the cell cycle. At 27 hpf, the proportion of dissociated *blm *cells in the G2/M phase (~31%) is approximately double that of wildtype siblings (~16%) consistent with defects in mitosis (Figure 3A). By immunohistochemistry, the total number of cells in the *blm *eye that express the G2/M marker phospho-histone H3 is lower than wildtype siblings (Figure 3B). However, consistent with the flow cytometry analyses (Figure 3A), the proportion of cells expressing the G2/M marker, normalized to the total number of nuclei, is significantly higher in *blm *mutants (8.7%) than in wildtype siblings (5.8%) (Figure 3C). At the transcript level, no significant difference is observed at 27 hpf for *p21-like *or *ccnb1*, G1 and G2 phase markers respectively, in *blm *mutants compared to siblings (Figure 3D). Equivalent levels of the post-mitotic retinal marker *atoh7 *were observed in *blm *and siblings indicating that retinogenesis had initiated in *blm *mutants (Figure 3D). With regards to cell death, a higher degree of apoptosis was observed in *blm *mutants at 24 hpf (Figure 3E). In summary, *blm *mutants exhibit an increased proportion of cells in G2/M phase, consistent with delayed cell cycle progression.

### Maternal *top2a *but not *top2b *is expressed pre-MZT

**Figure 4:**
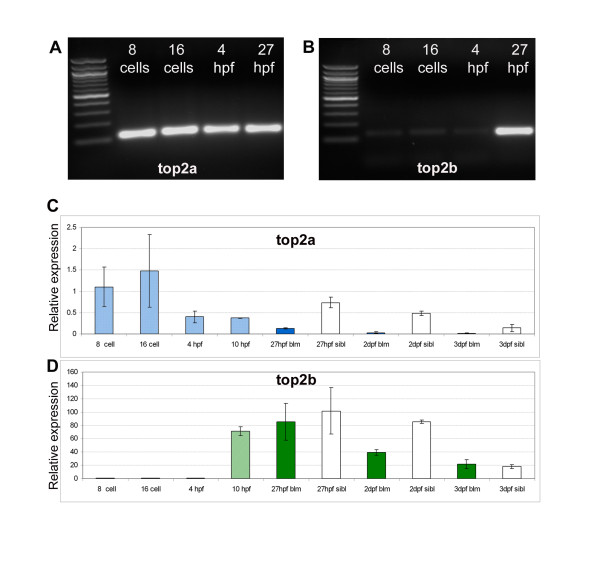
***top2a*, but not *top2b*, is expressed pre-MZT**. RT-PCR of wt embryos **(A-B) **and real time PCR of offspring from *blm *carriers **(C-D) **shows that maternal *top2a *(A, C), but not *top2b *(B, D), is present pre-MZT at 8- and 16-cell stages. *top2a *transcript levels decrease in *blm *siblings from 27 hpf to 3 dpf, and the expression in *blm *mutants is always significantly lower (C). The levels of *top2b *transcript also decrease in wildtype siblings from 27 hpf to 3 dpf, and although *top2b *levels in *blm *mutants and siblings is similar at 27 hpf, *top2b *is dramatically reduced in the mutants by 2 dpf (D). Error bars represent the standard error of the mean. At 8-cell, 16-cell, 4 hpf and 10 hpf stages mutant and wild type offspring are pooled. In graphs light blue (C) and light green bars (D) represent pooled wiltype and mutant embryos; dark blue/green bars represent *blm *mutants and white bars represent wildtype larvae.

The fact that cell cycle progression is occurring in *blm *larvae contrasts dramatically with the essential requirement of Top2a for mammalian embryo development beyond the 4-8 cell stage. Therefore, we hypothesised that *top2a^-/- ^*cells can proliferate in zebrafish *blm *mutants due to the presence of maternal *top2a *or functional redundancy with its *top2b *paralogue. The amino acid sequences of the zebrafish paralogues are 65% identical with the greatest divergence at the C-terminal end (Additional file 1). RT-PCR of RNA extracted from pre- (8 and 16 cell stages) and post-MZT (4 and 27 hpf) wildtype embryos demonstrates that maternal *top2a *transcript is abundant pre-MZT, whereas *top2b *transcript is not significantly expressed until after the onset of zygotic transcription (Figure 4A-B). Quantitative analysis by real-time PCR confirms these results (Figure 4C-D).

Real-time PCR also demonstrates a reduced level of *top2a *transcript in *blm *embryos compared to wildtype siblings at 1-3 dpf (Figure 4C). Zebrafish *top2b *transcript is negligible before 4 hpf, but is abundantly expressed at 10 hpf, a stage after MZT and before *blm *phenotypes are detected (Figure 4D). At 27 hpf, *top2b *levels are similar in *blm *mutants and widtype siblings. In siblings, *top2b *levels remain high at 2 dpf. In contrast, *top2b *levels decrease by ~50% in *blm *mutants by 2 dpf, consistent with the morphological defects observed at this developmental stage. In summary, maternal *top2a *could account for the extended pre- and/or post-MZT development of *blm *mutants whereas zygotic *top2b *could only account for the extended post-MZT development.

### No evidence of functional redundancy between *top2a *and *top2b in vivo*

**Figure 5:**
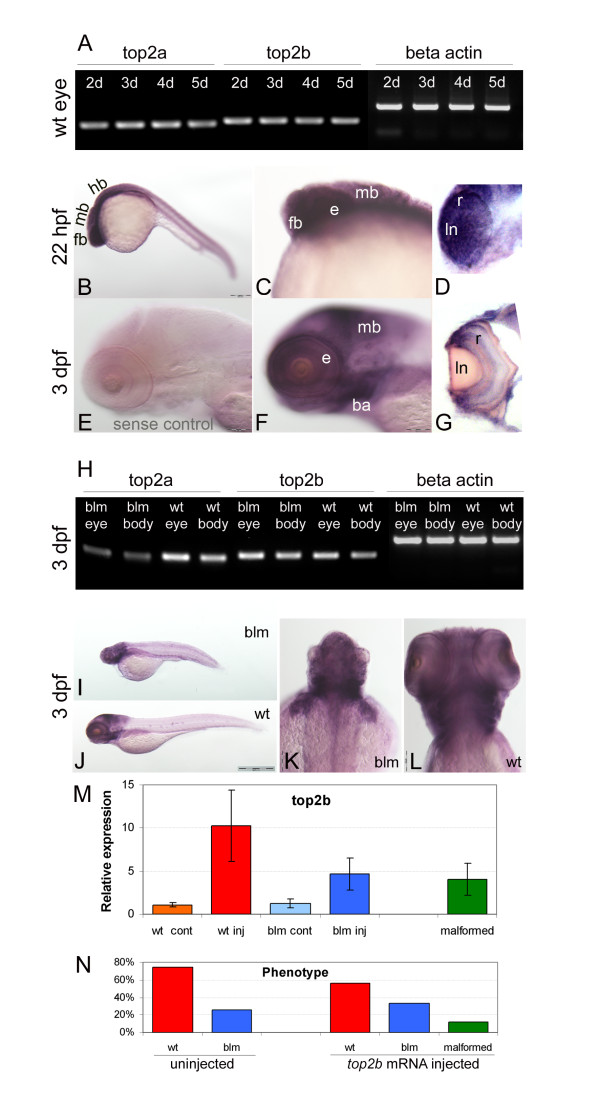
**Overlapping expression, but functional divergence of *top2a *and *top2b *paralogues *in vivo***. **A) **RT-PCR shows that both *top2a *and *top2b *are expressed in the eye at 2, 3, 4 and 5 dpf. **B-G) **Wholemount in situ hybridization reveals that *top2b *is expressed in the anterior of wildtype larvae including the forebrain, midbrain and eye (B-D) at 22 hpf and in the forebrain, midbrain, branchial arches and retina (F-G) at 3 dpf. E is the negative control sense probe; D and G are sections through the eye. **H) **RT-PCR at 3 dpf shows similar abundant expression of *top2b *in the eye and body of *blm *mutants and wildtype sibling. **I-L) **Wholemount in situ hybridization reveals a similar spatial expression pattern of *top2b *in *blm *(I, K) and wildtype larvae (J, L) at 3 dpf. **M) ***top2b *expression levels at 33 hpf following injection of *top2b *mRNA into 1-2 cell stage offspring of *blm *carriers. *blm *larvae overexpressing *top2b *RNA by 2.7-5.9 fold show no evidence of phenotypic rescue. N = 3 replicate experiments, n = 50 uninjected wild type (wt cont), n = 29 *top2b *RNA injected wild type (wt inj), n = 13 uninjected *blm *(blm cont), n = 17 *top2b *RNA injected *blm *(blm inj), n = 6 *top2b *RNA injected malformed. **N) **Phenotypes recorded at 33 hpf following injecting offspring of carriers of *blm *mutation with zebrafish *top2b *mRNA (N ≥ 3 replicate experiments, n = 117 uninjected wild type, n = 40 uninjected *blm*, n = 29 *top2b *RNA injected wild type, n = 17 *top2b *RNA injected *blm*, n = 6 *top2b *RNA injected malformed). Error bars represent the standard error of the mean. Abbreviations: ba - branchial arches, e - eye, fb - forebrain, hb - hindbrain, ln - lense, mb - midbrain, r - retina.

RT-PCR analyses confirm that *top2a *and *top2b *are co-expressed in the eye at 2-5 dpf (Figure 5A). Wholemount in-situ hybridisation in 22 hpf wildtype larvae reveals predominant zygotic expression of *top2b *in the forebrain and eyes, organs that display severe morphological abnormalities from 27 hpf in *blm *mutants (Figure 5B-D). At 3 dpf, *top2b *is expressed in the forebrain, midbrain, branchial arches and eyes (Figure 5F-G). T-PCR confirms that *top2b *is expressed in the *blm *eyes and body at 3 dpf (Figure 5H). Although the size of the eyes and head is reduced, the spatial expression of *top2b *in *blm *mutants at 3 dpf is not affected (Figure 5I-L). Overall, this suggests that *top2b *has biological potential to rescue affected tissues in *blm *mutants but cannot do so due to functional divergences between the *top2a *and *top2b *paralogues. In agreement, ectopic expression of *top2b *mRNA in *blm *mutants shows no evidence of rescuing the *blm *phenotype (Figure 5M-N and Additional file 2). Larvae overexpressing *top2b *by ~3.8 fold still exhibit the *blm *phenotype (Figure 5M). Morphologically, the average eye diameter of *blm *larvae overexpressing *top2b *is not significantly different from uninjected *blm *larvae (Additional file 2C). Thus, overexpression of *top2b *in *blm *larvae at non-toxic levels shows no evidence of rescuing the *blm *phenotype. In summary, our data suggests that *top2a *and *top2b *are not functionally redundant during embryo development.

### Chemical inhibition of Top2a delays development of pre-MZT embryos

**Figure 6:**
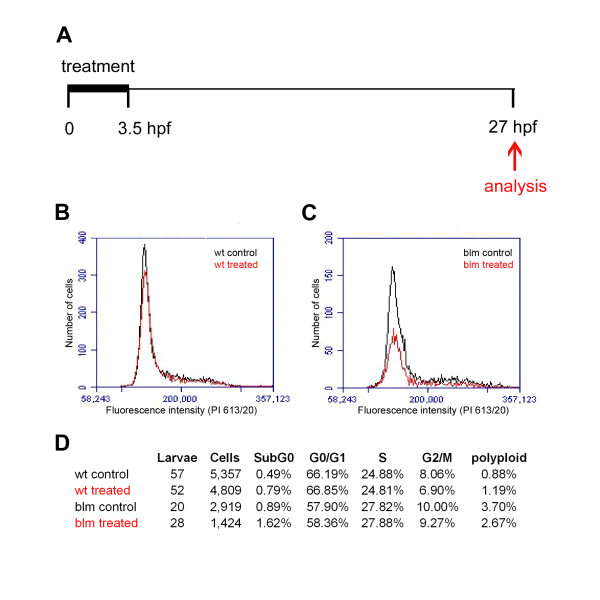
**Chemical inhibition of Top2a pre-MZT does not affect cell cycle profile at 27 hpf**. **A) **Schematic of the experimental procedure. 1-2 cell stage embryos were treated with 100 μM ICRF-193 for 3.5 hours when the drug was removed and the cell cycle profile analysed at 27 hpf. **B-C) **Flow cytometry analysis of cell cycle profiles of wt (B) and mutant (C) embryos. **D) **Table documenting the percentage of cells in cell cycle phases following ICRF-193 treatment.

To evaluate if maternal topoisomerase II proteins present in zebrafish embryos are required for early development, we chemically inhibited their function. ICRF-193 is a chemical inhibitor of TOP2A and TOP2B. With the goal of targeting the drug to maternal Top2a, and avoiding effects on zygotic Top2b, 1-2 cell stage embryos from matings of *blm *carriers were treated with ICRF-193 for ~3.5 hours, the drug removed before MZT and the embryos allowed to develop to 27 hpf. No discernible morphological defects were observed in ICRF-193 treated embryos compared to controls (Additional file 3). In replicate experiments, the cell cycle profile of treated embryos was analysed at 27 hpf (Figure 6A). Compared to their DMSO-treated controls, transient inhibition does not dramatically affect the percentage of sibling- or *blm*-treated cells in G_0_/G_1_, S, G_2_/M or polyploid categories (Figure 6B-D). A slight increase in the subG_0 _population of treated embryos is observed. Thus, transient treatment of pre-MZT embryos with a topoisomerase inhibitor causes no obvious morphological or cell cycle defects at a later developmental stage.

**Figure 7:**
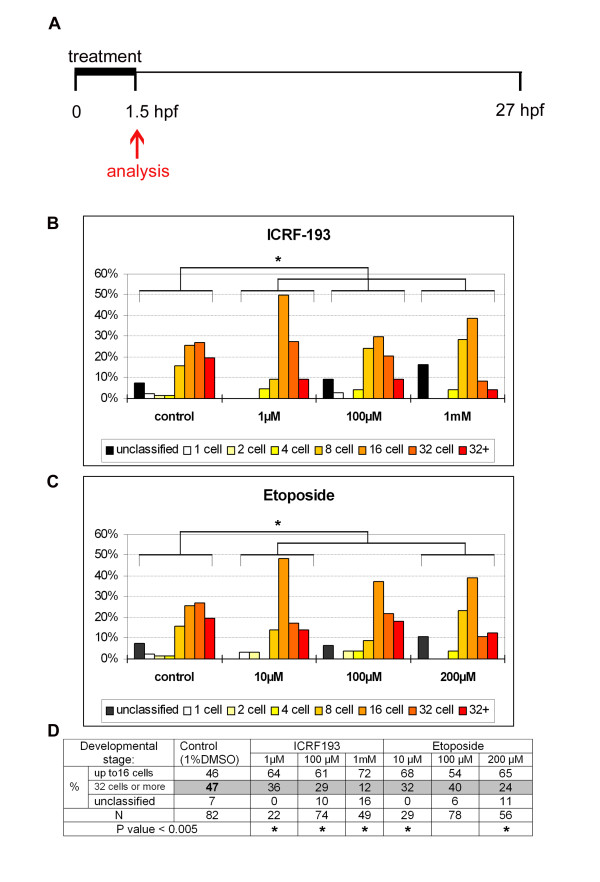
**Chemical inhibition of Top2a disrupts pre-MZT development of zebrafish embryos**. **A) **Schematic of the experimental procedure. 1-2 cell stage embryos were treated with Top2a inhibitors for 1.5 hours and their developmental progression was analysed. **B-C) **Graphs representing the percentage of embryos at a range of developmental stages following treatment with ICRF-193 (B) or etoposide (C). Top2a inhibition changes the staging distribution of embryos which peak at 16-32 cell stage in vehicle controls (DMSO) to peaking at the 8-16 cell stage in drug treated populations. The "unclassified" category includes embryos that do not fit into the standard developmental morphologies, ones in which all cells do not have typical optical transparency, and ones that are malformed. **D) **Table grouping the percentage of embryos at ≤ 16 cell stage or ≥ 32 cell stage for each drug treatment. Top2a inhibition increases the number of embryos ≤ 16 cell stage by up to 26%, and decreases the number of embryos ≥ 32 cell stage by up to 35%. Asterisks represent statistically significant differences (p < 0.005) to DMSO controls.

In an alternative approach (Figure 7A), focusing specifically on earlier cell divisions, zebrafish embryos at the 1-2 cell stage were treated for ~1.5 hours with a topoisomerase II inhibitor (ICRF-193) or poison (etoposide) and their developmental progression quantified according to established developmental staging series (Additional file 4). In embryo medium alone or in the presence of a drug not targeting topoisomerase II (L-AP4, a metabotropic glutamate receptor agonist), ~60-70% of embryos are staged at 16 cells (Additional file 5). In vehicle-treated controls (1% DMSO), performed concurrently with siblings, embryos are distributed equivalently across the 8-32+ cell stages (Figure 7B-C). In contrast, in ICRF-193 and etoposide-treated siblings there is an altered distribution peaking in the 8-16 cell stages (Figure 7B-C). These differences are statistically significant (p < 0.005) when compared to DMSO controls (Figure 7B-C). 1 mM ICRF-193 demonstrates a ~26% increase in the numbers of embryos ≤ 16 cell stage, and up to a ~35% reduction in the number of embryos > 16 cell stage (Figure 7D). The reduced number of cell divisions in over one quarter of the population is significant, considering the short treatment duration, the necessity for the drugs to penetrate the chorion, and the very high concentration of Top2a in zebrafish embryos (Figure 4), [29]. In summary, we conclude that maternal Top2a is required for the normal rate of embryonic cell division in developing zebrafish.

### Inhibition of *Xenopus *Top2a results in an extended M-phase in cycling extracts

**Figure 8:**
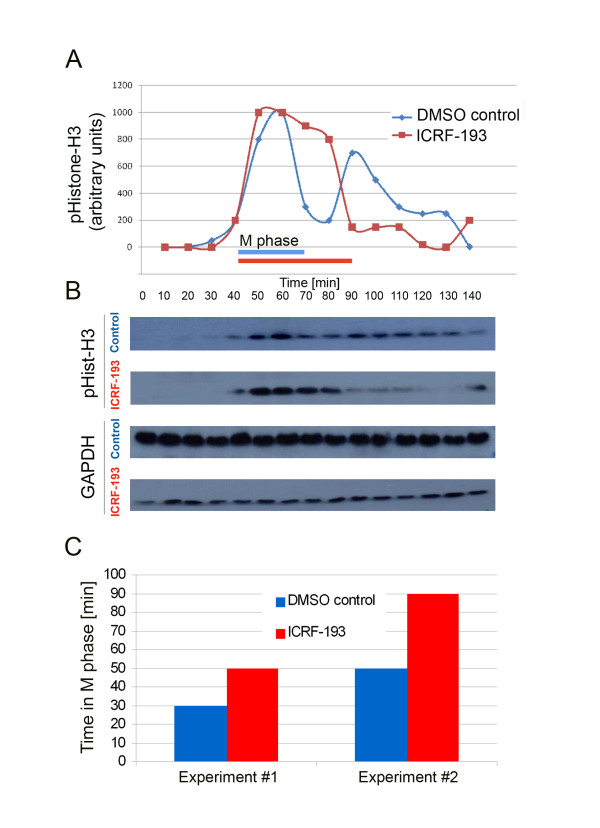
**Inhibition of maternal Top2a in pre-MBT cycling extracts delays cell cycle progression by lengthening the M-phase**. **A) **Cycling extracts from activated *Xenopus *eggs were treated with 20 μM Top2a inhibitor ICRF-193 diluted in 1% DMSO. To monitor cell cycle progression samples were taken every 10 minutes and phospho-histone H3 expression relative to GAPDH expression plotted. Blue and red bars indicate M phase in DMSO control and ICRF-193 treated extracts, respectively. **B) **Western blot analysis of phospho-Histone H3 and GAPDH expression levels. **C) **The increase in M phase length in two different cycling extracts treated with ICRF-193. M phase lengthened on average by 40% (SD = 3.14).

To further analyze the effect of Top2a inhibition on pre-MZT cell cycle progression we utilized cycling extracts prepared from activated *Xenopus laevis *eggs. These extracts recapitulate, *in vitro*, the cell cycle of the intact fertilized egg, with extracts oscillating between S phase and mitosis as in pre-MBT embryos [36]. In control extracts the first mitotic peak of phosphohistone H3 expression was observed after 60 minutes, with a subsequent mitotic peak observed at 90 minutes, thus recapitulating the 30 minute cycling interval observed in whole embryos (Figure 8A). Addition of ICRF-193 to extracts at time 0 resulted in a much longer M phase compared to control extracts (Figure 8A-B). Results from two different cycling extracts indicate a ~40% lengthened M phase following topoisomerase II inhibition (Figure 8C). Additionally, there was no evidence of a subsequent mitotic cycle in the following 50 minutes. In summary, we conclude that inhibiting maternal Top2a with ICRF-193 delays early embryonic cell cycle progression in M phase and may interfere with subsequent S phase entry.

## Discussion

### Top2a mutagenic susceptibility

Vertebrate topoisomerase II alpha genes appear particularly susceptible to somatic and germline mutations. Amplifications of the human *TOP2A *locus is associated with somatic mutations in subsets of breast, bladder and gastric cancers [6]. Notably, three genetically-distinct zebrafish *top2a *mutant alleles have now been uncovered (*hi3635*, *can4*, *blm*) in independent mutagenesis screens, suggesting that the *top2a *locus is also particularly susceptible to germline DNA perturbations [28,29]. All zebrafish *top2a *mutants display equivalent gross morphological phenotypes: small eye and brain, CNS necrosis, abnormal tail curvature and death at 4-5 dpf. Although, no further characterisation of the *hi3635 *insertion mutant has been reported, *can4 *and *blm top2a^-/- ^*alleles display similar cell cycle defects, including accumulation of mutant cells in G2/M and altered phosphohistone H3 expression (Figure 3), [29].

### The *blm *phenotype

**Figure 9:**
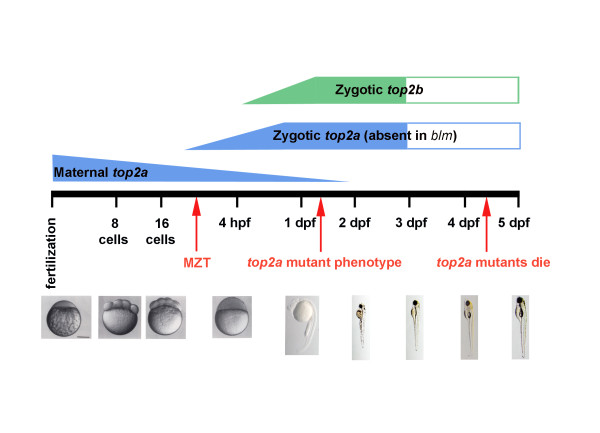
**Model depicting how maternal top2a enables pre-MZT development of zebrafish embryos and post-MZT development of *top2a *mutants**.

Our interpretation of the *blm *mutant phenotype is as follows (Figure 9). A nonsense mutation in the zebrafish *top2a *gene results in the expression of non-functional Top2a protein post MZT in *blm *mutants. The lower RNA expression levels may be due to lower transcription or increased decay of mutant *top2a *mRNA. The extensive embryonic and larval development of *blm *mutants arises from maternal expression of *top2a*, and not *top2b *which is negligible pre-MZT. Depletion of the maternal *top2a *transcript and Top2a protein results in the morphological phenotype of *blm *mutants at 27 hpf. Intracellular levels of *top2a*/Top2a diminish as daughter cells divide and as mRNA/protein degrades. For example, after only 10 divisions the level of *top2a *per daughter cell is expected to deplete by > 1000 fold relative to DNA content. Thus, tissues that are actively proliferating in later stages of development (*e.g*. eye) are preferentially affected. In those cells, Top2a depletion would likely perturb DNA decatenation during replication resulting in genomic instability and cell cycle checkpoint activation. The augmented levels of G_2_/M and apoptotic cells observed in *blm *mutants at ~27 hpf are consistent with activation of the known G2/M decatenation checkpoint present in adult cells leading to apoptosis.

Overall our data lead us to conclusion that zebrafish Top2a and Top2b are not functionally redundant *in vivo*. The zebrafish paralogs are ~65% identical at the protein level; both containing N-terminal ATP-binding sites and a central domain tyrosine required for DNA cleavage and ligation (Additional file 1). Only the C-terminal domains, containing nuclear localization sequences and phosphorylation sites, are highly divergent [8]. Previous studies in human cell lines suggested that *TOP2B *can compensate for depleted *TOP2A *[37,38]. In contrast, abundant zebrafish *top2b *transcript at stages post-MZT is not sufficient to prevent the *blm *phenotype, despite extensive temporal expression in tissues that develop morphological abnormalities in *blm *mutants. The onset of the *blm *phenotype correlates with depletion of maternal *top2a*, at a stage when zygotic *top2b *is abundant, and we identify embryos overexpressing ectopic *top2b *that retain the *blm *phenotype. Furthermore, in contrast to *top2a *mutants, the recently described *top2b *mutant displays a distinct phenotype in post-mitotic cells and not in proliferating cells [30]. There is a potential compensatory role for maternal Top2b in pre-MZT stages that we cannot directly exclude. However, pre-MZT *top2b *expression is negligible [33] and we have not found evidence of maternal deposition of Top2b in the literature. Notably, the functional divergence we observe *in vivo *between zebrafish Top2a and Top2b is in agreement with other in vitro studies reporting that the unique cell cycle functions of the Top2a isoform are dependent on specific C-terminal sequences [5,12,38]. Overall, our *in vivo *studies support that *top2a *and *top2b *do not exhibit complete functional redundancy during zebrafish embryo development.

### Role of maternal Top2a pre-MZT

Previous genetic approaches to analyse maternal *top2a *function using morpholino knockdowns have proven unsuccessful, probably because of the abundance of *top2a*/Top2a [29]. Therefore, to confirm that maternal *top2a *enables pre-MZT development, we pharmacologically targeted maternal Top2a protein using ICRF-193, a topoisomerase II catalytic inhibitor and etoposide, a topoisomerase II poison [11].

Our data supports the conclusion that maternal *top2a *is needed for the normal progression of pre-MZT embryos through developmental divisions. Both ICRF-193 and etoposide delay the progression of embryos through hierarchical developmental stages. This delay is reversible as washing out the drug results in the vast majority of zebrafish embryos proceeding beyond the 24 hpf, segmentation stage. It is likely that we are only partially inhibiting Top2a protein as complete inhibition is expected to arrest development earlier.

To explore the mechanism underlying the impaired developmental cell divisions in ICRF-193 treated embryos we utilised *Xenopus *cycling extracts which recapitulate *in vitro *the cell cycle of pre-MBT embryos [36]. Only topoisomerase II α is expressed in *Xenopus *eggs and embryos as demonstrated by the fact that depletion of Top2a from egg extracts abolishes topoisomerase II activity [39]. Our molecular analysis of *Xenopus *extracts treated with ICRF-193 indicates a lengthening of M phase. This is consistent with the previous morphological studies that observed anaphase stalling of pre-MZT zebrafish treated with etoposide [40].

We propose that the mechanism underlying delayed pre-MZT development and M phase lengthening following Top2 inhibition reflects activation of a cell cycle decatenation checkpoint. In the pre-MZT embryos lacking gap phases, Top2a inhibition results in a stoichiometric decrease in DNA decatenation during cell division activating a DNA decatenation checkpoint [40,41]. The M phase lengthening observed in ICRF-193 treated cycling extracts are consistent with defects in chromosome condensation and segregation most likely slowing the cycle and possibly activating a checkpoint in mitosis.

Inhibition of Top2a could be impacting on one or more phases of the cell division cycle and its effects in the simple early embryonic cell cycle and later somatic cycles where gap phases are present may be the result of different cell cycle processes being impeded. ICRF-193 is known to activate a G2 decatenation checkpoint in adult cells [42,43]. Interestingly, Top2a, but not Top2b, is required for activation of the decatenation checkpoint [43]. Catalytically inhibited Top2a exposes a C-terminal phospho-serine that recruits MDC1 to chromatin and activates the decatenation checkpoint [42]. As shown by our data, etoposide, which induces DNA damage in adult cells, phenocopies ICRF-193 in pre-MZT embryos. The likely explanation is the absence of Top2b and the absence of a DNA damage checkpoint in pre-MZT embryos [44]. This also suggests that etoposide alters Top2a topology to activate the decatenation checkpoint. In summary, we propose that inhibition of maternal Top2a activates a DNA decatenation checkpoint that delays mitosis and results in delayed embryonic development.

### Phenotypic differences between mouse and zebrafish *Top2a *mutants

Why does maternal Top2a enable zebrafish *top2a^-/- ^*mutants to progress to stages comprising of hundreds of thousands of cells, whilst mouse *Top2a^-/- ^*knockouts fail to divide beyond the 4-8 cell stage? One possibility is that maternal *Top2a *is not expressed in mammalian embryos. This is not the case, as studies show that catalytically active Top2a is functional at the 1-cell stage in mouse embryos [45]. Alternatively, maternal topoisomerase II alpha may have differential stability in mice and zebrafish due to the timing of MZT which occurs between embryonic day 0.5 and 1.5 (2-cell stage) in mouse but between 2.75 and 4.75 hours after fertilization (from 1,024 cells stage) in zebrafish [34,35,46]. Although some maternal transcripts are still present in embryonic day 2.5 murine morulas [46], previous reports demonstrate that newly synthesised mouse *Top2a *at the 2-cell stage is essential for cell cycle progression [45].

## Conclusions

The ability of zebrafish *top2a^-/- ^*mutants to proceed through thousands of developmental cell divisions starkly contrasts with mammalian *Top2a^-/- ^*knockouts which fail to divide beyond the 4-8 cell stage [20]. Our analyses reveal that pre-MZT, maternal *top2a *is sufficient to enable zebrafish *top2a*^-/- ^mutants to progress through early development. Post-MZT, zebrafish *top2b *expression is insufficient to fully compensate for the absence of *top2a in vivo *and *blm *mutants die at ~5 dpf (Figure 9). Top2a inhibition or loss results in clear cell cycle defects in early embryonic cell cycles of both zebrafish and *Xenopus*, as well as in later somatic cell cycles where *blm *mutants show evidence of G2/M decatenation checkpoint activation.

## Methods

All animal experiments were conducted under licences from the Department of Health and Children (B100/3641; B100/3003) and according to protocols approved by the University College Dublin Animal Research Ethics Committee (AREC-P-08-54; AREC-P-10-68).

### Mutagenesis

Zebrafish males of AB strain were mutagenised with ENU and mated to wildtype females to generate F_1 _founder fish [47]. F_1 _founders were mated to wildtype fish to establish 370 F_2 _families. F_3 _offspring of random incrosses within 200 F_2 _families were screened for recessive mutations at 5 dpf. 25% of the offspring from family Y-008 carriers presented with *blm *phenotypes.

### Light microscopy

Larvae at 27 hpf, 2 dpf and 3 dpf were euthanized with benzocaine and fixed in Sorensen phosphate buffer (pH 7.3) containing 4% PFA and 2.5% glutaraldehyde. Larvae were embedded in Epon resin and semi-thin (900 nm) sections stained with toluidine blue were photographed using a Leica DMLB microscope and a Leica DFC 480 camera.

### Mapping

Carriers of *blm *(AB strain) mutants were map-crossed to wildtype Tubingen fish to generate hybrid carriers AB*/Tub. 96 *blm *and 96 normal siblings from map crosses were used for bulk segregation analysis. Linkage was found to chromosome 12 with the *blm *mutation flanked by Z-markers Z99217 and Z10806. Several new simple sequence repeat (SSR) markers were designed to further narrow the critical interval of which zC13B10-SSR1 was the closest (6/96 recombinants). Primers zC13B10-SSR1-F: CTCCAATCGAGAGTCCTCGT, zC13B10-SSR1-R: AGCTGAAGGCCTGCTGTAAA. The *top2a *gene was localised in the critical interval.

### Sequencing

RNA from 3 dpf *blm *larvae was extracted using the Qiagen RNeasy Mini Kit and cDNA was synthesised using the Invitrogen Superscript III First Strand Synthesis for RT-PCR system. Six sets of primers were designed to amplify the whole reading frame of zebrafish *top2a*. The causative point mutation was sequenced using the following primers: forward 5'TGTTGCGCTACTGACTCGAC, reverse 5'TGGCCAGTTATGATGGATGA.

### Complementation assay

Hi3635 carriers were obtained from ZIRC. Complementation matings of *blm^+/- ^*♀ × *hi3635^+/- ^*♂ and *blm^+/- ^*♂ × *hi3635^+/- ^*♀ were performed. Phenotypes were recorded at 2 and 3 dpf.

### Flow cytometry

*blm *and wildtype sibling larvae were collected at 27 hpf. Embryos were dechorionated, rinsed in PBS and incubated for 10 minutes at 37°C in trypsin (1 mg/ml) with DNase I (1 U/200 μl) followed by mechanical disruption with a 25 G needle. Trypsin inhibitor was added to a final concentration of 1 mg/ml. After centrifugation, the cell pellet was fixed with 70% ethanol. Cells were rinsed with PBS, treated with RNase (10 μg/ml) and DNA stained with propidium iodide (40 μg/ml). DNA content was analysed using a Coulter EPICS XL-MCL or Accuri C6 flow cytometer.

### Quantification of mitotic cells in the eye

Embryos at 27 hpf were fixed at 4°C overnight in 4% PFA, then dechorionated, dehydrated in methanol and stored at -20°C. Prior to immunostaining embryos were rehydrated in PBS, permeabilised for 5 min in proteinase K (20 μg/ml) and post-fixed in 4% PFA for 30 min. Larvae were treated with blocking solution (2% normal goat serum, 1% Triton X-100, 1% Tween-20 in PBS) for 60 min followed by incubation in the primary antibody: anti-phospho-histone H3 at 1:200 dilution (Upstate Biotech) for 20 hours. Primary antibody binding was detected with Cy3-conjugated goat anti-rabbit secondary antibody (dilution 1:200). Nuclei were counter stained with DAPI. Z-stack images of whole larval eyes (optical sections at 2 μm interval) were taken with Zeiss LMS 510 Meta laser confocal microscope (with 63 × objective). Images were analysed with Imaris v7.2.3 software. To quantify the proportion of mitotic cells, embryos at 27 hpf were fixed in PFA, cryoprotected and sectioned (12 μm thickness). Mitotic nuclei were labelled with anti-phospho-histone H3 antibody (Upstate Biotech) and counter stained with DAPI. Images of sections through the eyes, head and spinal cord (at least 3 per animal) were taken with Zeiss AxioImager M1 microscope (objective 100×) and all PH3 positive and DAPI labelled nuclei from the field (89.5 μm × 67.1 μm) were counted. The percentage of mitotic cells in 5 mutant and 5 wild type larvae was compared.

### Acridine orange staining

To visualise apoptotic cells at 24 hpf, dechorinated wildtype and *blm *larvae were incubated with 5 μg/ml acridine orange (Sigma) for 30 minutes and washed in embryo medium. Larvae were anaesthetised with tricaine and imaged with an Olympus SZX2-ILLT microscope using fluorescence lamp and GFP filter.

### RT-PCR and Q-RT-PCR

RNA was extracted using the Qiagen RNeasy Mini Kit from pools (15 to 70 individuals) of 8-cell, 16-cell, 4 hpf, 10 hpf, 27 hpf, 2 dpf and 3 dpf embryos or dissected eyes and rest of the body of 2, 3, 4 and 5 dpf larvae obtained from wildtype incrosses or *blm *carrier incrosses. cDNA was synthesised by reverse transcription after priming with random hexamers using the Invitrogen Superscript III system. Primers' sequences for cell cycle markers [41]: p21-likeF-CCGTAGACCATGAGGAGC; p21-likeR-GTCTCGTCCACTTCTTTCTTTC; ccnb1F-GAGTCACAGCAATAAACCAC; ccnb1R-AGGAAGGCTCAGACACAAC. Primers' sequences for *top2a*, *top2b *and βactin: top2aF-AACGAGACCATGCCTCACC; top2aR-CAAACCAGCCTCTTTCTTCG; top2bF-GCAGTTGGAGGAAACTCTGC; top2bR-AGCTTCACAGCCGCATCTAT; βactF-GAGAAGATCTGGCATCACAC; βactR-ATCAGGTAGTCTGTCAGGTC. Primers' sequences for atoh7 (ath5): atoh7F-CCGGAGAAGTTTGAGAGTGC and atoh7R- GCTCAGAGCCATCTGTAGGG. Real-Time PCR was performed with SYBR-Green chemistry. 18s rRNA amplification with TaqMan probe served as an internal reference. For the time course experiment an arbitrary value of 1 was assigned to the average expression at 8 cell stage and expression level at other time points was normalised to this denominator. Plots of quantitative RT-PCR are average from 2 or 3 replicas and error bars represent standard error of the mean.

### Wholemount in situ hybridization

Full length *top2b *cDNA was amplified with AccuPrime Taq polymerase (Invitrogen) using forward and reverse primers binding to 5'UTR and 3'UTR (5UTRzfTop2b-TCTGGCCACACACAATAGAAA, 3UTRzfTop2b- CCAATCAGTTTTCTGGACCAA) and cDNA (made with random hexamers from total RNA of 1 dpf old larvae) as template. It was cloned into pGEM vector and used for synthesis of antisense and sense digoxigenin labelled probes with DIG in vitro transcription kit (Roche).

Larvae were fixed in 4% PFA (overnight at 4°C). 3 dpf specimens were treated for 20 mins with a bleach solution (30% H_2_O_2_, 50% formamide, 20% 20× SSC) to remove pigmentation and permeabilised with proteinase K (10 μg/ml, 22 minutes). Probe hybridisation was carried out at 62.5°C (overnight), followed by a series of formamide/SSC washes at the same temperature. Detection of hybridised probe was performed by incubation (overnight at 4°C) with AP-conjugated anti-digoxigenin Fab fragments (1:5,000; Roche) followed by colorimetric detection (NBT/BCIP, Roche). Whole larvae were imaged under 100% glycerol using an Olympus SZX2-ILLT stereo zoom microscope. 30 μm sections of stained larvae embedded in Tissue Tek (Sakura) were imaged using Zeiss Axioplan2 microscope equipped with AxioCam HRc camera.

### Overexpression of *top2b*

The *top2b *coding region was amplified from cDNA of 1 dpf larvae using AccuPrime Taq polymerase (Invitrogen) and cloned into pGEM vector. This plasmid was used to synthesise full length *top2b *mRNA with the mMessage mMachine SP6 system (Albion Inc.). Offspring of *blm *carriers were microinjected at 1-2 cell stages with zebrafish *top2b *mRNA at 250 ng/μl (approximately 75 pg per embryo). Phenotypes were recorded at 33 hpf and eye diameter was measured with Cell^F ^software (Olympus Soft Imaging Solutions) after imaging with an Olympus SZX2-ILLT stereo zoom microscope. To verify overexpression of *top2b*, pools of embryos presenting with wildtype, *blm *or malformed phenotypes were collected at 33 hpf for RNA extraction. Q-RT-PCR was performed as described above (using primers top2bF-GCAGTTGGAGGAAACTCTGC and top2bR-AGCTTCACAGCCGCATCTAT). Expression of *top2b *mRNA in all samples was normalised to the expression level in uninjected wildtype controls.

### Drug treatment

For transient treatment freshly laid eggs from *blm *carrier incrosses were incubated in embryo medium containing 100 μM ICRF-193 (Sigma) or 1% DMSO for 3.5 hours at 28°C and then the drug was replaced by fresh embryo medium. At 27 hpf larvae with mutant and wildtype phenotypes were separated and used for cell cycle analysis by flow cytometry.

For pre-MZT treatment, freshly laid wildtype AB eggs were placed in multi-well plates. Excess embryo medium was removed and replaced by a drug solution. Drugs were diluted in 1% DMSO in embryo medium at final concentrations of: 1 μM, 100 μM and 1 mM of ICRF-193 (Sigma) and 10 μM, 100 μM and 200 μM of etoposide (Ebewe). Embryos were incubated with drug for ~1.5 hours and then fixed with 4% PFA. Control embryos were incubated in 1% DMSO. The percentage of embryos reaching each development stage was calculated. Statistical analysis was performed using the Chi-squared test in GraphPad Prism.

### Cell cycle extracts

Cycling extracts were prepared from unfertilized *Xenopus laevis *eggs activated using calcium ionophore A23187 (Sigma; C7522), as described in [36]. Extracts were treated with 20 μM ICRF-193 diluted in 1% DMSO or 1% DMSO alone at time zero. Samples were collected at 10 minute intervals and snap frozen in liquid nitrogen.

### Western blotting

Protein samples were separated according to their apparent molecular mass under denaturing conditions on NuPAGE 4-12% Bis-Tris Gel, 1.0 mm × 17 well gels (Invitrogen), and transferred to PVDF membrane. After blocking in 5% Bovine Serum Albumin (BSA) in PBS-0.1% Tween-20, membranes were incubated with either anti-phospho-Histone H3 (Ser10) antibody from Millipore to detect mitosis (Cat. # 06-570) or GAPDH antibody from Cell Signaling (#3900), followed by incubation with horse-radish peroxidase-conjugated secondary antibodies. Proteins were visualized using Enhanced Chemiluminescence Western Blotting Substrate (Pierce; #32106). Densitometry was performed using the ImageJ program http://rsbweb.nih.gov/ij/.

## Abbreviations

dpf: days post fertilization; hpf: hours post fertilization; MZT: mid-zygotic transition; TOP2A: topoisomerase 2 alpha; TOP2B: topoisomerase 2 beta.

## Authors' contributions

BNK, YA, RC, FVE conceived and performed the mutagenesis experiment; BSR and SMcL characterized morphology; BSR, JRW and SMcL mapped and identified the mutation, BSR analyzed developmental expression pattern and cell cycle, BSR and OOL performed in situ hybridization, BSR, SMcL and JOS conceived and performed the drug treatment experiment and staging of embryos, CH and LOS conceived and performed cycling extract experiments, BSR and BNK drafted the manuscript. All authors read and approved the final manuscript.

## Supplementary Material

Additional file 1 **Comparison of zebrafish Top2a and Top2b paralogues**. **A) **Phylogenetic tree of Top2a and Top2b paralogues of 9 vertebrate species including zebrafish and other 4 teleost species. Protein sequences were retrieved from NCBI and Ensembl data bases. TOP2A: NP_001058.2 (human), NP_001003834.1 (zebrafish), NP_035753.2 (mouse), NP_001082502.1 (frog), NP_990122.1 (chicken), ENSTRUP00000041924 (fugu), ENSORLP00000005530 (medaka), ENSTNIP00000003411 (tetraodon), ENSGACP00000011260 (stickleback). TOP2B: NP_001059.2 (human), NP_001038656.1 (zebrafish), NP_033435.2 (mouse), XP_002932456.1 (frog), NP_990413.1 (chicken), ENSTRUP00000017341 (fugu), ENSORLP00000010828 (medaka), ENSTNIP00000021338 (tetraodon), ENSGACP00000010617 (stickleback). They were aligned by NCBI Multiple Alignment tool. Phylogenetic tree was generated with Seaview4 software (PhyML v3.0.1). Two distinct branches for Top2a and Top2b paralogues are apparent indicating that origin of two paralogues in zebrafish does not result from the genome duplication in teleosts. **B) **Alignment of zebrafish Top2a and Top2b protein sequences shows 65% identity (shaded residues) with the greatest divergence occurring at the C-terminal end. Asterisk indicates a serine residue in zebrafish Top2a which is associated with activation of the decatenation checkpoint in mammals. Click here for file

Additional file 2 **Overexpression of *top2b in blm embryos***. Offspring of *blm *carriers were microinjected at 1-2 cell stages with zebrafish genomic *top2b *sequence (BAC clone CHORB736O22185Q) at 25 ng/μl (7.5 pg per embryo) or zebrafish *top2b *mRNA at 250 ng/μl (75 pg per embryo) and analysed at 33 hpf. **A) **Table of observed phenotypes upon injecting offspring of carriers of *blm *mutation with zebrafish *top2b *BAC clone or in vitro synthesised *top2b *RNA. **B) **Images of 33 hpf malformed larvae overexpressing *top2b *by ~4 fold following injecting with *top2b *mRNA. **C) **Ectopic expression of *top2b *mRNA does not rescue the small eye phenotype of *blm *embryos. Error bars represent the standard error of the mean. Click here for file

Additional file 3 **Transient inhibition of Top2a pre-MZT**. **A) **Dark field images showing the morphology of embryos at 27 hpf following treatment with ICRF-193 from 1-2 cell stage until 3.5 hpf **B) **Representative confocal images (projections of 30 slices taken at 0.5 μm intervals) of 3.5 hpf embryos stained with DAPI, which had been treated from 1-2 cell stage with 100 μM ICRF-193. Chromatin appears to be more compacted in the treated embryos but not extensive DNA damage was observed. Click here for file

Additional file 4 **Staging of embryos treated with topoisomerase inhibitors**. Top panel: images of normally developed embryos at 16, 32 and 64 cell stages. Bottom panels: example images of "not classified" or malformed embryos treated with 1% DMSO (vehicle control), ICRF-193 or etoposide. This group includes embryos with atypical shape: **A) **irregular shape, protruding animal pole, **B) **asymmetric animal pole and uneven cell size, **C) **irregular shape and opaque, **D-E) **undefined cell morphology and abnormal transparency, **F) **small, irregular-shaped animal pole, **G) **abnormal distribution of cells around yolk, **H) **undefined cell morphology with abnormal distribution around yolk, **I) **duplicated/split animal pole. Click here for file

Additional file 5 **Effect of topoisomerase inhibitors on pre-MZT development of zebrafish embryos is specific**. Treatment of 1-2 cell embryos with L-AP4 (metabotropic glutamate receptor agonist) does not change the distribution of developmental stages compared to untreated controls in embryo medium. Incubation in 1% DMSO (vehicle for topoisomerase inhibitors) increases the percentage of embryos in more advanced stages of development. **A) **Table and **B) **graphs representing the number and percentage, respectively of embryos at 8-32 cell developmental stages following incubation for 1 hour in embryo medium containing 10 μM, 200 μM or 1 mM L-AP4 (water dilutions), 1% DMSO or embryo medium alone. Click here for file
