# Supplementary figures and images for: Maternal topoisomerase II alpha, not topoisomerase II beta, enables embryonic development of zebrafish top2a-/- mutants
Source: BMC Dev Biol. 2011 Nov 23;11:71. doi: 10.1186/1471-213X-11-71 (PMC3287258; doi:10.1186/1471-213X-11-71)

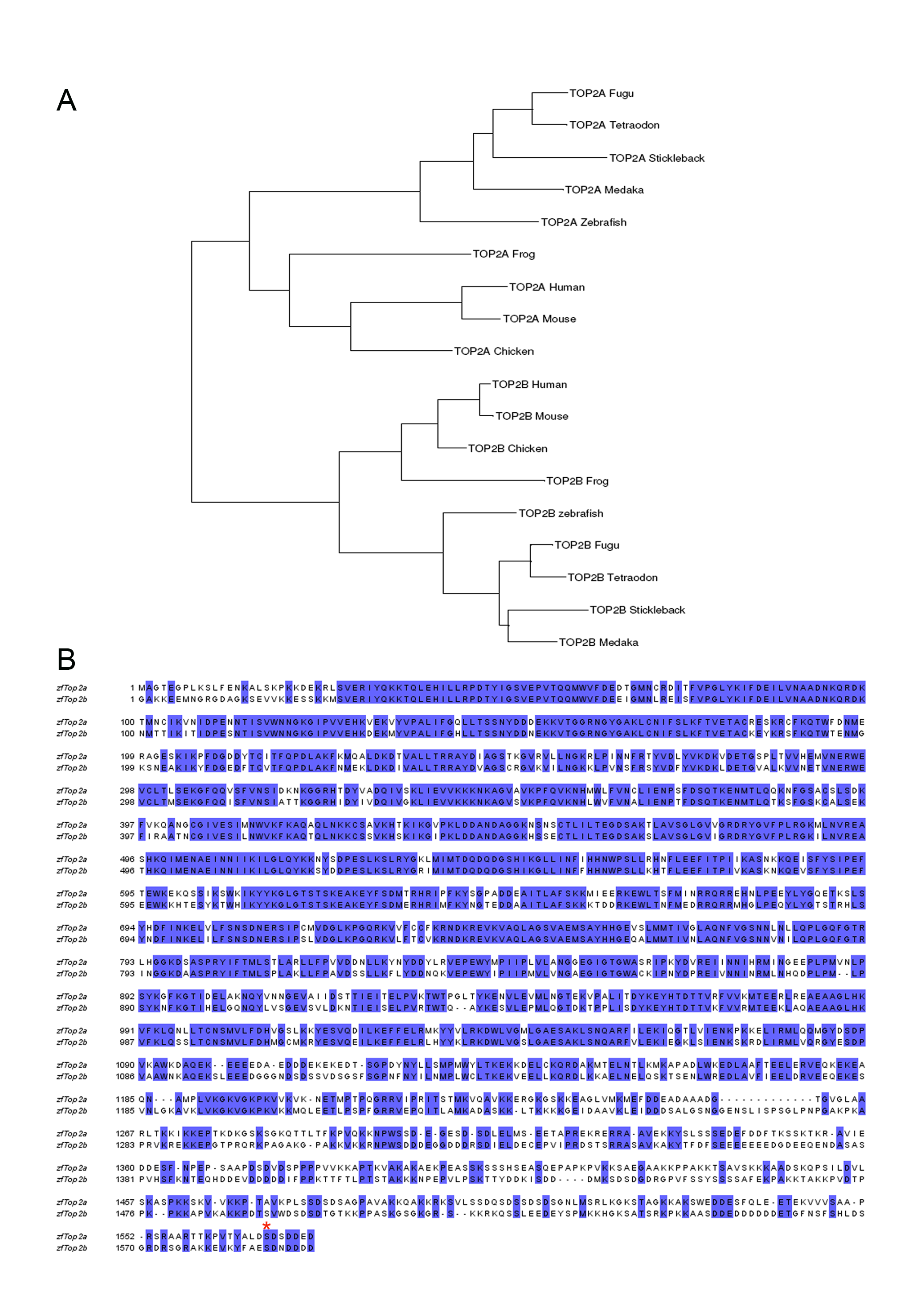

Supplement: Additional file 1 — Comparison of zebrafish Top2a and Top2b paralogues. A) Phylogenetic tree of Top2a and Top2b paralogues of 9 vertebrate species including zebrafish and other 4 teleost species. Protein sequences were retrieved from NCBI and Ensembl data bases. TOP2A: NP_001058.2 (human), NP_001003834.1 (zebrafish), NP_035753.2 (mouse), NP_001082502.1 (frog), NP_990122.1 (chicken), ENSTRUP00000041924 (fugu), ENSORLP00000005530 (medaka), ENSTNIP00000003411 (tetraodon), ENSGACP00000011260 (stickleback). TOP2B: NP_001059.2 (human), NP_001038656.1 (zebrafish), NP_033435.2 (mouse), XP_002932456.1 (frog), NP_990413.1 (chicken), ENSTRUP00000017341 (fugu), ENSORLP00000010828 (medaka), ENSTNIP00000021338 (tetraodon), ENSGACP00000010617 (stickleback). They were aligned by NCBI Multiple Alignment tool. Phylogenetic tree was generated with Seaview4 software (PhyML v3.0.1). Two distinct branches for Top2a and Top2b paralogues are apparent indicating that origin of two paralogues in zebrafish does not result from the genome duplication in teleosts. B) Alignment of zebrafish Top2a and Top2b protein sequences shows 65% identity (shaded residues) with the greatest divergence occurring at the C-terminal end. Asterisk indicates a serine residue in zebrafish Top2a which is associated with activation of the decatenation checkpoint in mammals. [file 1471-213X-11-71-S1.TIFF]

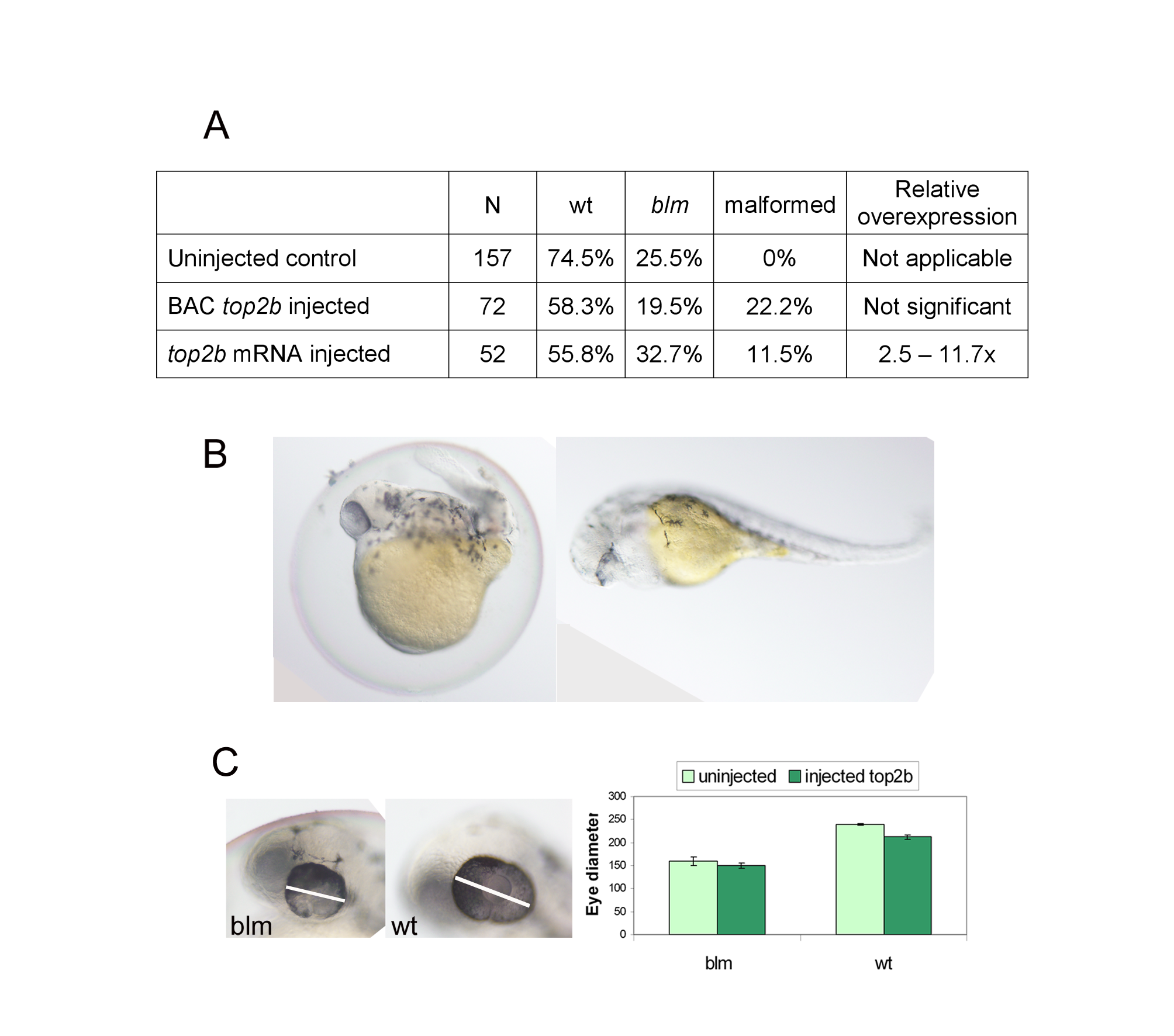

Supplement: Additional file 2 — Overexpression of top2b in blm embryos. Offspring of blm carriers were microinjected at 1-2 cell stages with zebrafish genomic top2b sequence (BAC clone CHORB736O22185Q) at 25 ng/μl (7.5 pg per embryo) or zebrafish top2b mRNA at 250 ng/μl (75 pg per embryo) and analysed at 33 hpf. A) Table of observed phenotypes upon injecting offspring of carriers of blm mutation with zebrafish top2b BAC clone or in vitro synthesised top2b RNA. B) Images of 33 hpf malformed larvae overexpressing top2b by ~4 fold following injecting with top2b mRNA. C) Ectopic expression of top2b mRNA does not rescue the small eye phenotype of blm embryos. Error bars represent the standard error of the mean. [file 1471-213X-11-71-S2.TIFF]

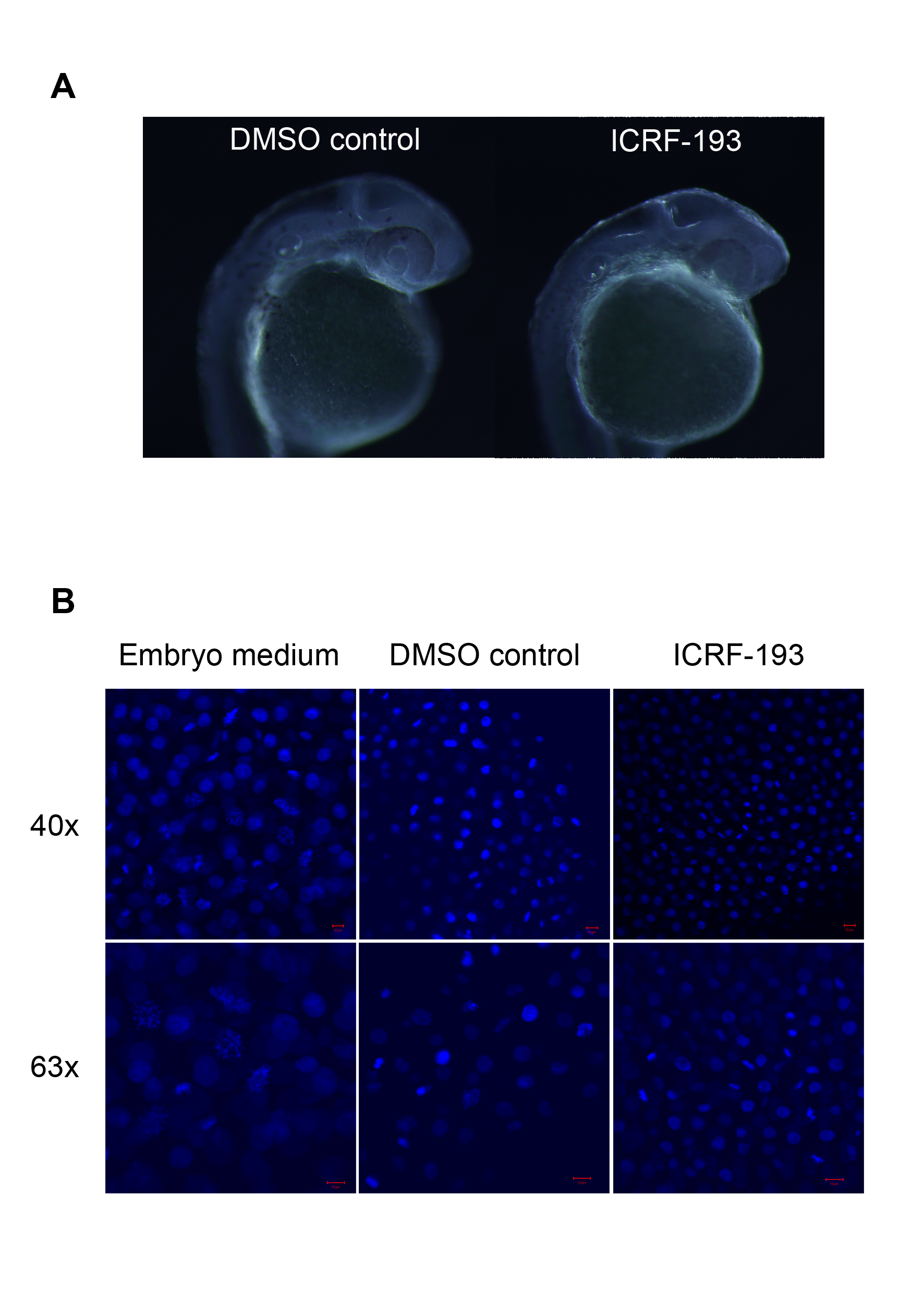

Supplement: Additional file 3 — Transient inhibition of Top2a pre-MZT. A) Dark field images showing the morphology of embryos at 27 hpf following treatment with ICRF-193 from 1-2 cell stage until 3.5 hpf B) Representative confocal images (projections of 30 slices taken at 0.5 μm intervals) of 3.5 hpf embryos stained with DAPI, which had been treated from 1-2 cell stage with 100 μM ICRF-193. Chromatin appears to be more compacted in the treated embryos but not extensive DNA damage was observed. [file 1471-213X-11-71-S3.TIFF]

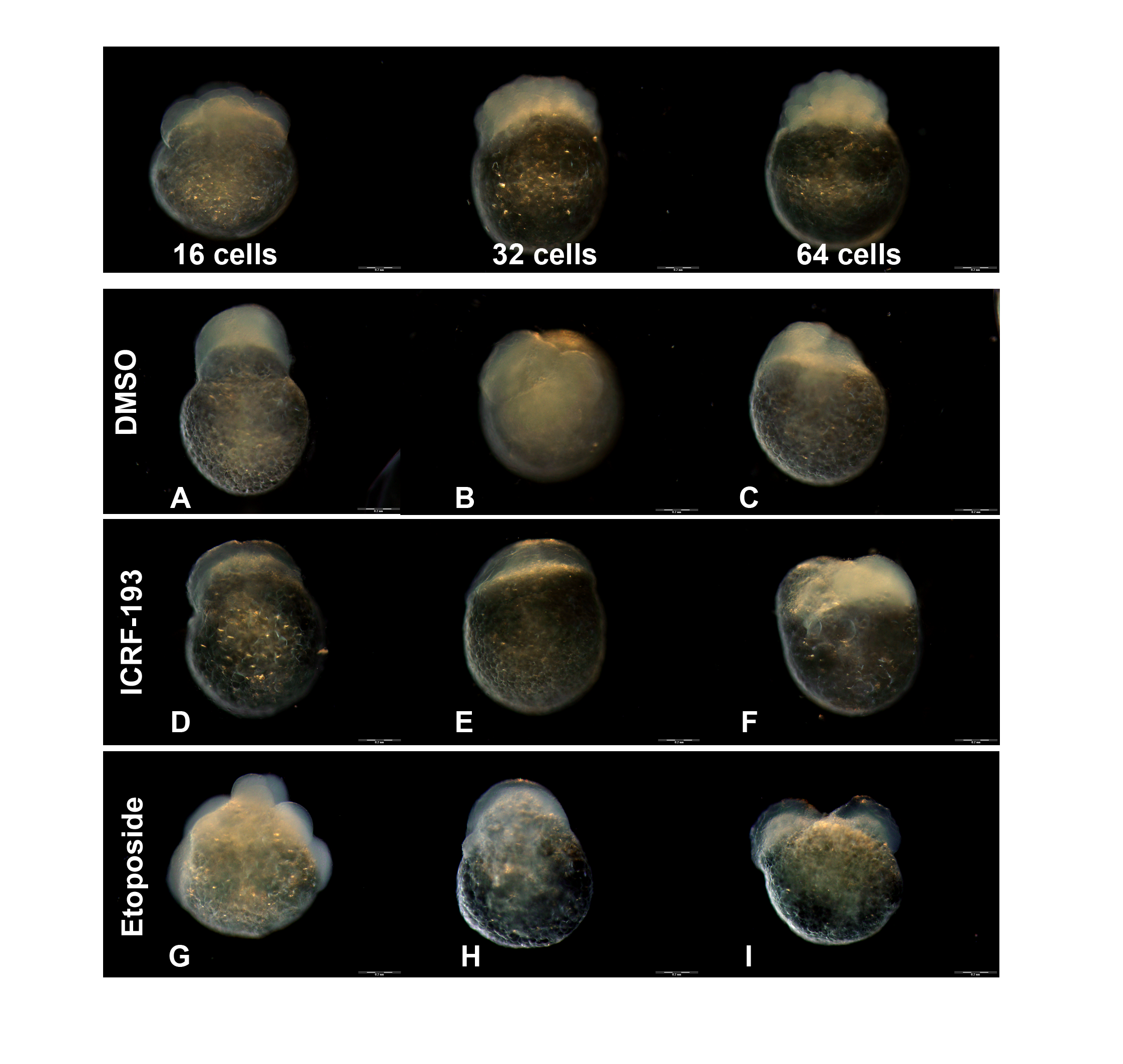

Supplement: Additional file 4 — Staging of embryos treated with topoisomerase inhibitors. Top panel: images of normally developed embryos at 16, 32 and 64 cell stages. Bottom panels: example images of "not classified" or malformed embryos treated with 1% DMSO (vehicle control), ICRF-193 or etoposide. This group includes embryos with atypical shape: A) irregular shape, protruding animal pole, B) asymmetric animal pole and uneven cell size, C) irregular shape and opaque, D-E) undefined cell morphology and abnormal transparency, F) small, irregular-shaped animal pole, G) abnormal distribution of cells around yolk, H) undefined cell morphology with abnormal distribution around yolk, I) duplicated/split animal pole. [file 1471-213X-11-71-S4.TIFF]

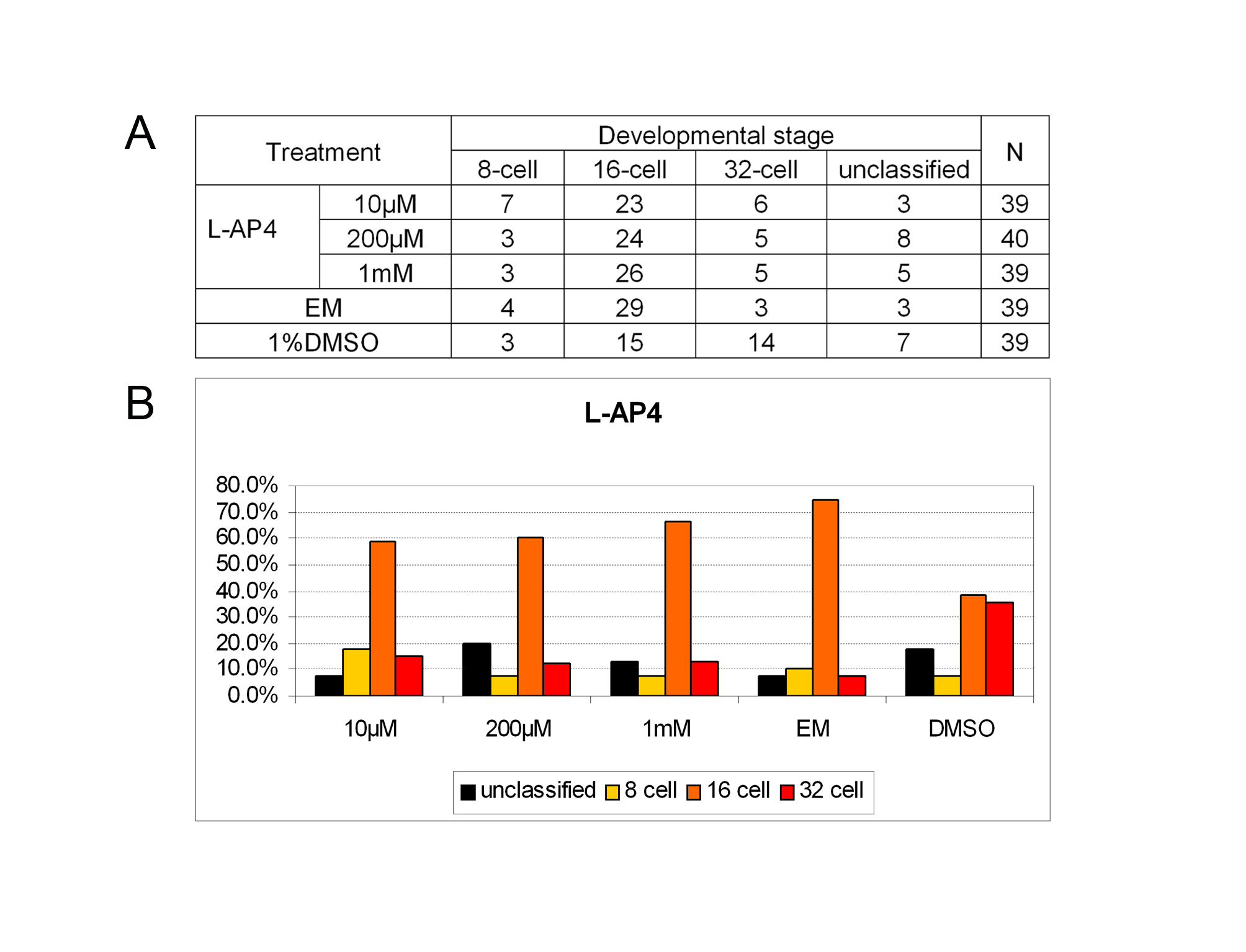

Supplement: Additional file 5 — Effect of topoisomerase inhibitors on pre-MZT development of zebrafish embryos is specific. Treatment of 1-2 cell embryos with L-AP4 (metabotropic glutamate receptor agonist) does not change the distribution of developmental stages compared to untreated controls in embryo medium. Incubation in 1% DMSO (vehicle for topoisomerase inhibitors) increases the percentage of embryos in more advanced stages of development. A) Table and B) graphs representing the number and percentage, respectively of embryos at 8-32 cell developmental stages following incubation for 1 hour in embryo medium containing 10 μM, 200 μM or 1 mM L-AP4 (water dilutions), 1% DMSO or embryo medium alone. [file 1471-213X-11-71-S5.TIFF]
